# Volatilomics as an Emerging Strategy to Determine Potential Biomarkers of Female Infertility: A Pilot Study

**DOI:** 10.3390/biomedicines10112852

**Published:** 2022-11-08

**Authors:** Ana Teresa Brinca, Ofélia Anjos, Maria Manuel Casteleiro Alves, Ângela Sousa, António Hélio Oliani, Luiza Breitenfeld, Luís A. Passarinha, Ana Cristina Ramalhinho, Eugenia Gallardo

**Affiliations:** 1 Health Sciences Research Centre, Faculty of Health Sciences, University of Beira Interior, 6200-506 Covilhã, Portugal; 2 Instituto Politécnico de Castelo Branco, Quinta da Senhora de Mércules, 6001-909 Castelo Branco, Portugal; 3 CEF—Centro de Estudos Florestais, Instituto Superior de Agronomia, Universidade de Lisboa, Tapada da Ajuda, 1349-017 Lisboa, Portugal; 4 Assisted Reproduction Laboratory of Academic Hospital of Cova da Beira, 6200-251 Covilhã, Portugal; 5 São José do Rio Preto School of Medicine, Gynaecology and Obstetrics, São José do Rio Preto 15090-000, Brazil; 6 C4—Cloud Computing Competence Centre, University of Beira Interior, 6201-001 Covilhã, Portugal; 7 Applied Molecular Biosciences Unit, Departament of Chemistry, NOVA School of Science and Technology, Universidade NOVA de Lisboa, 2829-516 Caparica, Portugal; 8 Associate Laboratory i4HB-Institute for Health and Bioeconomy, NOVA School of Science and Technology, Universidade NOVA de Lisboa, 2819-516 Caparica, Portugal; 9 Laboratório de Fármaco-Toxicologia, UBIMedical, University of Beira Interior, 6200-284 Covilhã, Portugal

**Keywords:** infertility, polycystic ovary syndrome, endometriosis, premature ovarian failure, follicular fluid, volatile organic compounds, solid-phase microextraction, gas chromatography–mass spectrometry

## Abstract

Due to its high prevalence, infertility has become a prominent public health issue, posing a significant challenge to modern reproductive medicine. Some clinical conditions that lead to female infertility include polycystic ovary syndrome (PCOS), endometriosis, and premature ovarian failure (POF). Follicular fluid (FF) is the biological matrix that has the most contact with the oocyte and can, therefore, be used as a predictor of its quality. Volatilomics has emerged as a non-invasive, straightforward, affordable, and simple method for characterizing various diseases and determining the effectiveness of their current therapies. In order to find potential biomarkers of infertility, this study set out to determine the volatomic pattern of the follicular fluid from patients with PCOS, endometriosis, and POF. The chromatographic data integration was performed through solid-phase microextraction (SPME), followed by gas chromatography–mass spectrometry (GC-MS). The findings pointed to specific metabolite patterns as potential biomarkers for the studied diseases. These open the door for further research into the relevant metabolomic pathways to enhance infertility knowledge and diagnostic tools. An extended investigation may, however, produce a new mechanistic understanding of the pathophysiology of the diseases.

## 1. Introduction

Infertility, or impaired fecundity, is defined as the inability to generate a clinical pregnancy after six months to one year of unprotected sexual activity [1,2]. Around 186 million people, both women and men, are impacted globally with this medical condition [3,4]. The World Health Organization (WHO) recognized infertility as a social disease when it became clear that it posed a threat to contemporary reproductive medicine [3,5], becoming a public health issue [6,7]. Infertility is a disease affecting couples, since individuals might be in excellent physical and mental condition but still be unsuccessful in conceiving as a pair [1,3,5,6,8,9]. Age, acute or chronic illnesses, environmental contaminants, occupational exposures, general lifestyle choices, infectious diseases, genetic abnormalities, and specific reproductive disorders can affect either men or women and impact fertility rates [6]. Due to the social environment, women tend to seek medical attention and establish reproductive health evaluations at an earlier age [1]. Ovarian dysfunction, fallopian tube obstruction, and irregular uterine shape are the typical causes of female infertility [1]. When approaching such cases, the diagnosis should be targeted to make the process less time-consuming and more effective and achieve the best possible chances for a safe pregnancy and posterior delivery [3]. The prevalence of infertility has remained consistent over the past few decades [6,8,9]. However, the demand for infertility services has significantly increased, partly because of trends toward delayed childbirth and improvements in assisted reproductive technology [6,8,9]. Clinical behavior varies greatly due to the many conditions that cause infertility and the body’s reaction to hormonal stimulation. Therefore, in addition to the currently available diagnostic and treatment tools, different approaches must continue to be developed in order to improve the success of infertility treatments [10].

PCOS is one of the prevailing gynecological, endocrine, and metabolic disorders among women of reproductive age, representing one of the leading causes of anovulatory infertility. The Rotterdam Consensus on Diagnostic Criteria for PCOS estimates that 5% to 20% of women globally are affected by this condition [11,12,13,14]. In order to be diagnosed with PCOS, two out of the following three traits must be present: biochemical or clinical hyperandrogenism (HA) [15], oligoanovulation [16], and polycystic ovarian morphology [17]. These women usually present metabolic abnormalities [18,19,20,21] that can further generate an increased risk of type 2 diabetes [19,22,23], insulin resistance [19,24,25], and cardiovascular diseases [19,26,27,28,29,30] and disturb the body mass index [18,19]. Endometriosis is a complex disease that affects 10–15% of reproductive-age women [31,32], 10–50% of infertile women, and approximately 80% of women suffering from chronic pelvic pain [33]. Endometriosis is a condition in which ectopic tissue with glands and stroma, such as the endometrium, grows outside the uterine cavity [31,33,34,35,36,37]. This abnormality is a gynecologic disease associated with estrogen-dependent chronic inflammation [31,33,34,38,39]. It leads to reproductive dysfunction, infertility, and the development of chronic pelvic pain syndrome [31,33,38]. POF occurs when the exhaustion of the number of ovarian follicles is concurrent with autoimmune ovarian damage, along with genetic predisposition [40,41], and develops in about 1% of women [40,41]. Due to ovarian disfunction, its main features are oligomenorrhea or frequent menstruation; blood with increased gonadotropin and follicle-stimulating hormone (FSH) levels; decreased AMH, antral follicle count (AFC), inhibin B, estradiol, testosterone, and estrogen levels; and a series of low-estrogen symptoms [40,42,43,44,45,46,47,48,49].

Germ cell–somatic cell communication is possible due to follicular fluid (FF), a complex milieu. It includes a range of metabolites and allows for several reactions essential to oocyte development [12,50]. It is composed of metabolites produced in the follicular wall, transudates of plasma, and serum diffusion. Additionally, substances derived from regional follicular metabolic processes and ovarian cell biological functions, such as granulosa and theca cells, also contribute to and change FF composition according to the oocyte’s needs. Therefore, the oocyte’s growth and differentiation in vivo are directly linked to this biological matrix [50,51,52,53]. Further research into conditions such as PCOS, endometriosis, and early ovarian failure will benefit from a thorough knowledge of FF, since it is a non-invasive matrix that provides biological insights into fertility, reflecting the changes in the patient’s microenvironment. FF chemical and biomolecular hallmarks have sparked a great deal of attention, becoming a crucial source of knowledge and inspiring much research to find new targets for assessing oocyte development. Therefore, a detailed characterization and consideration of FF may identify potential metabolites that interfere with normal female function and promote infertility [50,52,53,54].

Metabolomics, a high-throughput technique, identifies various metabolic components in diverse biological matrixes. Untargeted metabolomics focuses on the dynamic adjustment of small molecules in response to an organismal disturbance, offering significant insights into the pathophysiology of various diseases and helping identify specific biomarkers [12,50]. These molecules are present in biological matrixes such as tissues, cells [10,50,55,56], blood, urine, plasma, serum, and FF [10,50,51,55,56,57] due to their medical and chemical signature, which allows for the quantitative measurement of the dynamic chemical reactions that take place in living systems in response to pathophysiological insults or genetic variations [10,50,55,56]. Volatilomics, a metabolomic subgroup that reflects biochemical metabolic activity and environmental influences, offers new insights into the physiological processes of several disorders [58,59,60,61]. It encompasses all volatile organic compounds (VOCs) that derive from exogenous sources, including nutrition, medications, and environmental exposure, as well as internal sources, such as endogenous metabolomic and biochemical processes [58,59,60]. Multiple pathologies, such as cancer, genetic and metabolic disorders, schizophrenia, and infectious diseases, have already been connected to particular VOC signatures [59,62] and potential biomarkers [63,64]. The volatilomics approach is based on highly sensitive analytical techniques and does not require invasive procedures, since VOCs can be found in readily accessible biofluids [61,65,66,67], such as urine [61,63,66,68,69,70,71,72], exhaled breath [61,63,66,71,72,73,74,75,76,77], saliva [61,63,71,72,78,79,80,81,82,83], blood [63,71,72], serum [61,78,79,83], skin emanations, breast milk [63,71,72], and tissues [10,69,84,85]. Volatilomics studies involve the targeted analysis of a limited number of metabolites connected to a specific biological pathway or the fingerprinting of a significant fraction of metabolites [61,65,66,67]. In this study, a more untargeted approach was required to detect and identify unexpected changes in the concentrations of specific metabolites [61,86].

Instrumentation that is precise, dependable, and efficient is essential for VOC detection [61,68]. Due to its consistent and reproducible results, headspace (HS) solid-phase microextraction (SPME) in combination with gas chromatography–mass spectrometry (GC-MS) is the typically used method for the extraction and posterior analysis of VOCs [10,61,69,84,85,87,88]. SPME involves the partitioning of analytes from the sample solution into the sorbent coating of the SPME fiber due to the intermolecular interaction with the sorbent material [61,87,88,89]. This method is very effective, since it combines sampling, extraction, and concentration, resulting in greater sensitivity, automation, and portability while reducing the concentration of interferents [61,69,87,90,91]. Mass spectrometry (MS) is the most widely used technique for determining the volatilomic profile of biological matrices. It separates metabolites by gas or liquid chromatography, followed by ionization and resolution according to the mass-to-charge ratio. High sensitivity and low-concentration secondary metabolite detection are two main features of MS techniques [61,65,92]. GC is now one of the most widely used techniques for quantifying and qualifying multicomponent mixtures, showing the best separation power. The combination of the two techniques results in improved sensitivity, specificity, and the separation of the components to be analyzed [93,94,95]. It also enables the determination of detailed information regarding the structure of various compounds, allowing the correct identification and quantification based on their mass-to-charge ratio (*m*/*z*) [93,96].

This study aimed to qualitatively determine all the VOCs present in the FF of women diagnosed with diverse causes of infertility. Subsequently, and according to the results obtained, we traced the metabolomic profile of each clinical condition, compared the control FFs and FFs referring to the different pathologies, and established possible biomarkers. This research identified 136 VOCs, and 37 (27%) of them were found in at least two samples. To our knowledge, this is the first study to attempt to establish the volatomic profile of the FF from women with endometriosis, PCOS, and POF who underwent IVF procedures and find possible biomarkers for these diseases using HS-SPME/GC-MS combined with multivariate statistical tools. This high-throughput methodology may be applicable to clinical settings as a diagnostic approach or as a means to improve diagnostic decisions when combined with the existing diagnostic and screening tools.

## 2. Materials and Methods

### 2.1. Materials and Reagents

The SPME fiber holder for manual use and the 100 μm polydimethylsiloxane (PDMS) coated fibers were obtained from Supelco (Bellefonte, PA, USA). The SPME fibers were conditioned according to the manufacturer’s instructions.

### 2.2. Subject Sample Collection

To investigate the metabolomic pattern of FF, 52 samples from women who underwent IVF procedures were analyzed. These include 15 patients with PCOS, 8 with endometriosis, 12 with POF, and 17 controls. The 17 controls corresponded to women submitted to IVF procedures due to specific conditions that did not affect the FF, such as tubal obstruction, or when the couple’s primordial fertility factor was male-driven. Women were enrolled between October 2015 and July 2019. All subjects were Caucasian. The samples were obtained at the Assisted Reproduction Laboratory of the Academic Hospital Center of Cova da Beira in Covilhã, Portugal. All experiments were performed in accordance with the standard guidelines and national requirements, namely, the Declaration of Helsinki and Portuguese Law 21/2014, and approved by the institutional ethics committee of the Academic Hospital Center of Cova da Beira, Covilhã, Portugal (reference number 47/2015, approved on 15 July 2015). Informed consent was obtained from all individuals before inclusion.

### 2.3. Follicular Fluid Sample Collection

Oocyte retrieval was performed by transvaginal ultrasound-guided aspiration 36 h after the injection of human chorionic gonadotrophin, and each follicle was aspirated. To avoid any blood contamination, only clear fluid samples were included, whereas bloodstained and cloudy follicular fluid samples were excluded.

### 2.4. Follicular Fluid Preparation

All FF samples from the same patient were pooled, and a volume of 15 mL was centrifuged at 3 kg for 15 min. Supernatants were filtered with 0.2 um filters to eliminate cell debris and then stored at −80 °C until cfDNA extraction.

### 2.5. Extraction of Metabolites from the FF

**Figure 1:**
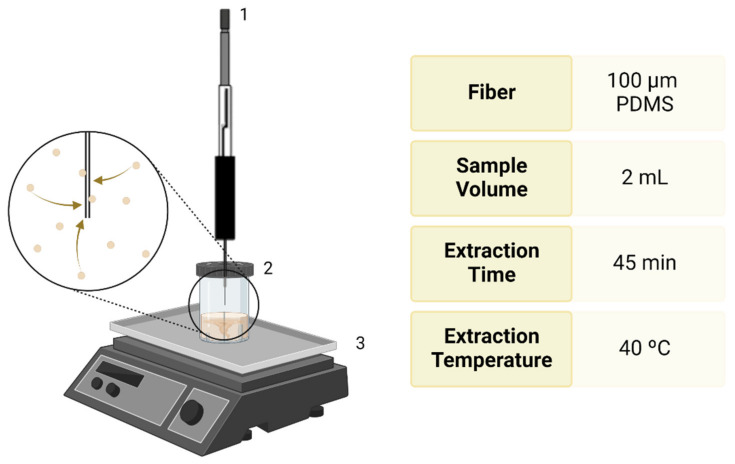
Schematic representation of VOC extraction from FF: (1) SPME syringe; (2) vial with 2 mL of FF; (3) heating mantle with magnetic stirrer. Created using BioRender.com.

During the procedure, all samples were stored at 4 °C. After placing 2 mL of each FF in a vial, the volatile metabolites were extracted using a 100 μm PDMS, non-bonded SPME fiber exposed in the headspace of the flasks for 45 min at 40 °C, under continuous agitation (125 rpm). This procedure is diagrammed in Figure 1. Subsequently, the SPME syringe was injected into the GC injection port for 5 min to allow the desorption of VOCs from the fiber. This methodology was adapted from a previous study carried out by C. Silva et al. [97]. PDMS fibers were chosen due to their compatibility with volatile analytes ranging from 80 to 500 MW and compatibility with a manual holder.

### 2.6. Gas Chromatography–Mass Spectrometry (GC-MS) Conditions

VOCs in the headspace were analyzed using an HP 7890B gas chromatographic system in conjunction with an Agilent Technologies 5977A mass spectrometer and an Agilent 7693 autosampler. For the separation of the analytes, a capillary column (30 m, 0.25 mm I.D., 0.25 m film thickness) with 5% phenylmethylsiloxane (HP-5MS) was provided by J & W Scientific (Folsom, CA, USA). The oven temperature profile was: (a) 5 min at 45 °C; (b) increase in temperature until 150 °C, at a rate of 2 °C min^−1^; (c) 150 °C for 10 min; (d) increase in temperature until 220 °C, at a rate of 7 °C min^−1^; and (e) 220 °C for 10 min. Column flow was kept constant at 1.0 mL/min using helium (He ultrapure, Nippon gases, Vila Franca de Xira, Portugal) as the carrier gas. The injection port was maintained at 250 °C and operated in the splitless mode (5 min). Regarding MS analyses, the operating temperatures of the transfer line, quadrupole, and ionization source were 280, 150, and 230 °C, respectively. The electron impact mass spectra were recorded at 70 eV, the ionization current was 35μ A, and data acquisition was performed in scan mode (50–550 *m*/*z*). The identification of metabolites was performed by comparing mass spectra using Agilent MS ChemStation software, version B.04.03 (Palo Alto, CA, USA) equipped with the NIST20, Wiley12, and SWGDRUGv8 mass spectral libraries with a similarity threshold higher than 80%, or using commercial standards when available.

## 3. Results and Discussion

A heatmap was obtained, representing values for the main variable of interest across two axes as a cluster effect. To create the heatmap, STATISTICA 7 (StatSoft. Inc., Tulsa, OK, USA) software was used.

**Figure 2:**
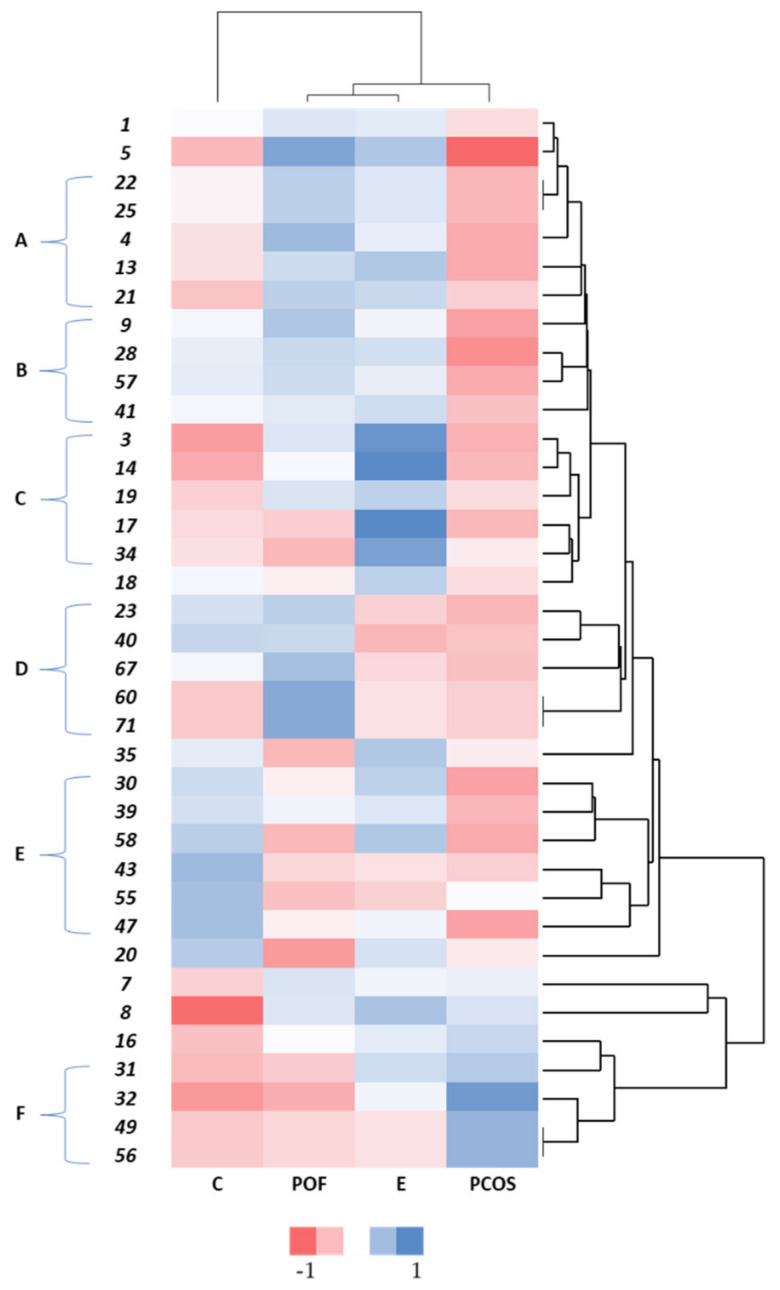
Heatmap of the correlation and tendencies between the FFs of the four medical conditions and respective VOCs. The abscises correspond to: (C) controls; (POF) premature ovarian failure; (E) endometriosis; (PCOS) polycystic ovary syndrome. The coordinates represent the VOCs considered, which were assigned numbers. They were separated into six groups according to their distribution throughout the samples. Each number corresponded to a single VOC: (1) palmitic acid; (3) tetradecanal; (4) 2,4-di-tert-butylphenol; (5) diethyl phthalate; (7) 1,2,3,4-tetramethylbenzene; (8) 4-methyl-2,4-bis(4-hydroxyphenyl)pent-1-ene; (9) palmitic acid ME; (13) isopropyl myristate; (14) octadecanal; (16) tetradecamethylcycloheptasiloxane; (17) hexadecanal; (18) gamma-stearolactone; (19) dodecane; (20) dodecamethylcyclohexasiloxane; (21) hexadecyloxirane; (22) octadecane; (23) diisooctylphthalate; (25) 1,2,3,5-tetramethylbenzene; (28) stearyl alcohol; (30) stearic acid; (31) tetradecamethylhexasiloxane; (32) hexadecamethylheptasiloxane; (34) eicosamethyl-cyclodecasiloxane; (35) octadecan-1-ol trimethylsilvyethe; (39) cyclotetradecane; (40) hexadecanoic acid; (41) methyl stearate; (43) heptadecane; (47) butyl-2-methylpropylphthalate; (49) ethyl xylene; (55) tetracosamethyl-cyclododecasiloxane; (56) docosane; (57) hexamethyldisiloxane; (58) 1,3-di-tert-butylbenzene; (60) 1-dodecanol; (67) oleamide; (71) 4,6-dimethyldodecane.

Figure 2 represents a heatmap of the different metabolites and their respective tendencies towards each disease. From this heatmap, it is possible to observe some associations between the samples and their unique metabolomic expressions. The colours range from red to blue according to the comparative abundance of metabolites in the FFs. To compare the results for each pathology, red indicates a lower presence, while blue indicates a greater presence.

**Figure 3:**
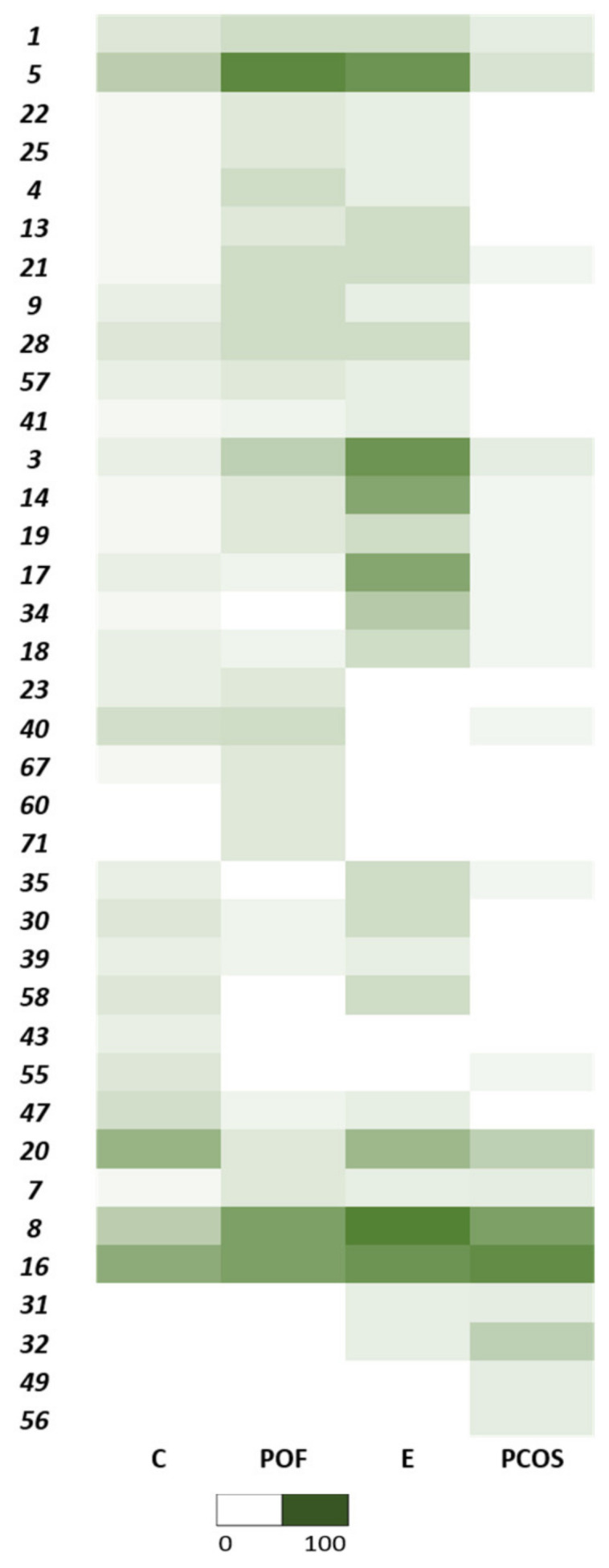
Percentual occurrence of VOCs in all the samples for each medical condition. The abscises correspond to: (C) controls; (POF) premature ovarian failure; (E) endometriosis; (PCOS) polycystic ovary syndrome. The coordinates represent the VOCs considered, which were assigned numbers: (1) palmitic acid; (3) tetradecanal; (4) 2,4-di-tert-butylphenol; (5) diethyl phthalate; (7) 1,2,3,4-tetramethylbenzene; (8) 4-methyl-2,4-bis(4-hydroxyphenyl)pent-1-ene; (9) palmitic acid ME; (13) isopropyl myristate; (14) octadecanal; (16) tetradecamethylcycloheptasiloxane; (17) hexadecanal; (18) gam-ma-stearolactone; (19) dodecane; (20) dodecamethylcyclohexasiloxane; (21) hexadecyloxirane; (22) octadecane; (23) diisooctylphthalate; (25) 1,2,3,5-tetramethylbenzene; (28) stearyl alcohol; (30) stearic acid; (31) tetradecamethylhexasiloxane; (32) hexadecamethylheptasiloxane; (34) eicosamethyl-cyclodecasiloxane; (35) octadecan-1-ol trimethylsilvyethe; (39) cyclotetradecane; (40) hexadecanoic acid; (41) methyl stearate; (43) heptadecane; (47) bu-tyl-2-methylpropylphthalate; (49) ethyl xylene; (55) tetracosamethyl-cyclododecasiloxane; (56) docosane; (57) hexamethyldisiloxane; (58) 1,3-di-tert-butylbenzene; (60) 1-dodecanol; (67) oleamide; (71) 4,6-dimethyldodecane.

Figure 3 depicts a percentual representation of all VOCs present for each medical condition. Several VOCs had similar incidences throughout the various health conditions, such as palmitic acid, tetradecamethylcycloheptasiloxane, cyclotetradecane, and methyl stearate. At the same time, some metabolites were not present in all the samples pertaining to a specific pathology. Therefore, some metabolites could not be identified with a particular medical condition.

The controls, represented by “C”, showed a profile that differentiated itself from those of the infertility complications. The most frequently identified metabolites were tetradecamethylcycloheptasiloxane, with an occurrence of 59%; dodecamethylcyclohexasiloxane (53%); 4-methyl-2,4-bis(4-hydroxyphenyl)pent-1-ene (35%); and diethyl phthalate (35%). Even though tetradecamethylcycloheptasiloxane was present in several control samples, it is notable that this compound was also present in the remaining samples from women with clinical conditions associated to infertility. Additionally, these FFs presented a small incidence of several compounds. Metabolites such as 1-dodecanol, 4,6-dimethyldodecane, and all VOCs identified in group F were not represented.

POF and E were the two diseases with the most similarities, forming a cluster of their own. Diethyl phthalate, a phthalic acid ester, was found in 83% of the endometriosis samples and 75% of the POF samples. This metabolite was identified in samples of both these conditions twice as frequently as in the controls. Phthalic acid esters are considered endocrine disrupters [98,99,100,101]. Some toxicological studies have described phthalates as toxic for human reproduction [102,103,104]. They decrease fertility rates [105], produce anti-androgenic effects by reducing testosterone and estrogen production at high doses [106,107,108], affect folliculogenesis [105] by restricting antral follicle growth through the inhibition of 17-beta-estradiol production [109,110,111,112], generate ovarian dysfunction [109,110,111,112], and impact oocyte maturation and embryonic development [105]. Du et al. found relevant phthalate concentrations in the FF of women undergoing IVF [105]. These can arise from intoxication, industrial exposure, or ingestion [72,113,114]. In 2013, Upsona et al. released a study on the exposure to select phthalates, showing how pervasive they were among female enrolees. Their research also suggested that phthalates may increase the risk of developing endometriosis, a hormonally mediated disease, among reproductive-age women [115]. Buck Louis et al. also demonstrated that select phthalates are associated with a higher chance of an endometriosis diagnosis [106]. Furthermore, these compounds can be found in distinct biological matrixes and are related to diseases such as cancer [72,113,114]. However, there is still a lack of consistency in these results, reducing the impact of some findings and methodologies [106,115,116,117,118,119,120,121,122].

The POF samples presented exclusive compounds, 1-dodecanol and 4,6-dimethyldodecane. Even though they were only present in 17% of the samples, as shown in Figure 3, Figure 2 demonstrates a relevant correlation when considering the controls and remaining diseases. The compound 1-dodecanol appeared in the para-axillary and nipple–areola regions of pregnant women. Some reports have shown that this VOC is affected by emotional anomalies [123]. When compared to the controls, urinary samples from specific types of cancer also presented high levels of 1-dodecanol, namely colorectal cancer, leukaemia, and lymphoma [124]. However, its effects on the reproductive system are not fully understood, and more accurate data are required to formulate a precise mechanism of action for this metabolite [125]. Groups A and B were also highly represented in the POF FFs, as exemplified by diethyl phthalate. On the other hand, group E was mainly absent, as shown by the VOC dodecamethylcyclohexasiloxane.

Endometriosis had a versatile volatilomic profile, with specific compounds detected in many samples. Since these metabolites were not as abundant in the other samples, they might be able to characterize the disease. Endometriosis samples were most obviously correlated with metabolites from group C (tetradecanal (75%), octadecanal (63%), hexadecanal (63%), and eicosamethyl-cyclodecasiloxane (38%)). Group C components varied throughout all the samples, but the FFs from the three pathologies presented a slight prevalence of these metabolites compared with the controls. However, according to the heatmap, these may not be suitable markers. Octadecanal and tetradecanal are both fatty aldehydes [126,127]. Tetradecanal, also known as myristyl aldehyde, is the reduced form of myristyl acid [127]. Hexadecanal is a volatile straight-chain aldehyde [72] and a final product of glycosphingolipid metabolism [128]. Its metabolization forms phospholipids that can produce signals within cells [129,130]. Hexadecanal is present in several biological fluids [72], but its levels tend to be low, particularly in cumulus–oocyte complexes, according to some animal studies [129]. Cordeiro and his team showed that age is closely related to enhanced glycosphingolipid metabolism [131], demonstrating a negative correlation. Overall, sphingolipids are associated with steroid hormone synthesis, mainly through the modulation of steroidogenic pathways. These molecules may act as secondary messengers or paracrine regulators for genetic transcription, although the sphingolipid mechanism is still not fully understood [132,133]. The abundance of these compounds in FF may indicate alterations in the proper steroidogenesis process for these patients [132]. Sphingolipid breakdown is also a relevant event during apoptosis [131,133,134]. Tetradecamethylhexasiloxane and hexadecamethylheptasiloxane, belonging to group F, and some metabolites from group A, such as diethyl phthalate, might also help differentiate the FF of women with endometriosis. The first two are also siloxanes [135], and tetradecamethylhexasiloxane has already been related to male infertility [136,137]. Dodecamethylcyclohexasiloxane (cyclomethicone 6) is a cyclic dimethyl polysiloxane compound [138], which was mostly found in the FF of controls and endometriosis patients. Its toxicity is confirmed, and some studies have reported that odecamethylcyclohexasiloxane causes endometrial tumours. Its mechanism of action, however, remains a mystery [139].

Marianna et al. used 1H-NMR to analyze the FF of women with various stages of endometriosis. When compared to the endometriosis patients, the follicular fluids of the controls contained lower levels of phospholipids, lactate, and insulin and higher levels of fatty acids, lysine, choline, glucose, aspartate, alanine, leucine, valine, proline, phosphocholine, and total LDH [140]. According to NMR data collected by Karaer and coworkers, the metabolomic profiles of the follicular fluids from women with endometriosis contained higher levels of glucose, pyruvate, and valine, as well as higher concentrations of lactate, unlike the studies carried out by Marinna et al. [141]. LysoPC (18:2(9Z,12Z)) and LysoPC (18:0) were upregulated, in line with Sun et al.’s SWATH study, whereas phytosphingosine was downregulated [142].

The PCOS samples presented the smallest number of compounds. Even though the prevalent VOC was tetradecamethylcycloheptasiloxane, 1-ethyl-2,3-dimethylbenzene and docosane might be the best predictors, as they were only present in the FF of PCOS patients. Tetradecamethylcycloheptasiloxane, also known as cyclomethicone 7, is a cyclic dimethyl polysiloxane compound [138]. These metabolites are known to interfere with fertility and present potential carcinogenic effects (uterine tumours in females). They increase ovarian atrophy and vaginal mucification [143], disturb hormonal function, and are reproductive toxicants [144]. The compound 1-ethyl-2,3-dimethylbenzene, or ethyl xylene, is considered a BTEX (benzene, toluene, ethylbenzene, xylene) member [145]. Exposure to these components leads to several health concerns, especially regarding female reproduction and its regulators [146,147]. Human studies have demonstrated alterations in the menstrual cycle, abnormal endocrine function, adverse birth outcomes, and other potential reproductive health risks [148]. Furthermore, again considering Figure 3, the components from group F might also help differentiate the PCOS profile. The absence of group A and B components might also be used to characterize the PCOS profile, since these metabolites were relatively well-represented in the controls.

The metabolite 4-methyl-2,4-bis(4-hydroxyphenyl)pent-1-ene was very abundant in the controls, but its occurrence was even higher in the samples of the three diseases. This discrepancy may be of high relevance. The compound 4-methyl-2,4-bis(4-hydroxyphenyl)pent-1-ene, or MBP, is also a phthalate metabolite [105]. It was present in 88% of the endometriosis samples and 67% of both the PCOS and POF samples. It is formed by the liver S9 fractions, and its metabolic activation may occur in the fetal liver, being detected as an in vivo metabolite in the fetus [149]. MBP is a very potent estrogenic metabolite of bisphenol A (BPA) [149], an interferent for oocyte development and maturation [98]. However, it presents 1000 times more biological activity than BPA [150]. The binding to steroid receptors is one of many possible mechanisms that might lead to endocrine disturbance. The development and operation of the reproductive system rely on the androgen receptor (AR) and progesterone receptor (PR). The endogenous native AR and PR ligands may be blocked or interfered with by MBP, affecting the AR- and PR-mediated pathways and resulting in malfunction [150]. Okuda et al. demonstrated that MBP-potent estrogenic activity affected uterine weight, myometrial thickness, and luminal epithelial cell height in rat studies [151]. MBP activity has also been associated with breast cancer [152,153], lung disfunction [154], and pancreatic β-cell death [155].

Hou et al., using GC-MS analysis, showed that elevated L-tryptophan and L-tyrosine caused metabolic alterations in PCOS FF, and these complications in amino acid metabolism could negatively influence patients [156]. When compared to controls, the FF exhibited elevated concentrations of chenodeoxycholic acid-3-d-glucuronide, glycocholic, taurocholic, and glycochenodeoxycholic acids, suggesting that this metabolic pathway is also significantly affected by bile acid metabolism, according to Yang et al.’s analysis of this pathway employing ultra-performance liquid chromatography/tandem mass spectrometry [157]. Gongadashetti et al.’s findings revealed that the PCOS group’s levels of ROS, TAC, and 8-IP were higher than those of the controls [158]. Via high-performance liquid chromatography/mass spectrometry, several authors have discovered differences between species in the metabolism of lipids, such as triglycerides, phosphatidylethanolamines, and phosphatidylinositols [12,159,160]. Zhang and his team used NMR to show increased glycoprotein, acetate, and cholesterol and decreased levels of lactic acid, glutamine, pyruvate, and alanine in the FF, indicating a change in pyruvate metabolism and glycolysis [161].

Although the FF presents numerous biomarkers, there are few consistent results across the literature. The parameters utilized in the analytical procedures, such as the method of FF sample preparation, the presence of impurities, and the mass range, have generated disagreement. This may have arisen from the examination of follicles with various diameters and discrepancies in the measurement techniques, patient age, BMI, number of samples examined, ovarian stimulation, genetics, and other diagnosed diseases [11,13,132,158,162,163,164].

Our results exhibited interesting discriminating factors for FF samples, demonstrating that volatilomics could be an advantageous approach for identifying potential infertility biomarkers. Our findings also suggested the possibility of classifying certain endogenous metabolites.

## 4. Conclusions

In this work, we described the application of an HS-SPME/GC–MS methodology to determine the VOCs present in FF samples from women with clinical manifestations related to infertility. The GC-MS analysis identified 136 VOCs in all 52 specimens, corresponding to 15 PCOS, 8 endometriosis, and 12 POF patients and 17 controls. Due to their prevalence in all the samples, 37 of the 136 VOCs were studied, and multivariate statistical analysis revealed significant alterations in the levels of certain metabolites according to each pathology. The altered biochemical profiles revealed several compromised metabolomic pathways in the various diseases, with endometriosis and POF presenting several similarities. The high-throughput methodologies employed suggested the possibility of using metabolite identification as a springboard for determining potential infertility biomarkers. Our findings may also benefit the exploration of the associated metabolomic pathways and the improvement of clinical diagnostic tools. However, it should be noted that this research represents a pilot study, and more testing is needed regarding the volatilomic profile of FF in order to improve future prospects.

## Data Availability

Data are contained within the article.
